# A citizen science model for implementing statewide educational DNA barcoding

**DOI:** 10.1371/journal.pone.0208604

**Published:** 2019-01-10

**Authors:** Anthony Chiovitti, Frazer Thorpe, Christopher Gorman, Jennifer L. Cuxson, Gorjana Robevska, Christopher Szwed, Jacinta C. Duncan, Hannah K. Vanyai, Joseph Cross, Kirby R. Siemering, Joanna Sumner

**Affiliations:** 1 Gene Technology Access Centre (GTAC), The University High School, Parkville, Victoria, Australia; 2 School of Biosciences, University of Melbourne, Victoria, Australia; 3 Texas A&M University, College Station, Texas, United States of America; 4 Australian Genome Research Facility (AGRF), Victorian Comprehensive Cancer Centre, Melbourne, Victoria, Australia; 5 Museums Victoria, Carlton Gardens, Victoria, Australia; Eugene Lang College of Liberal Arts at the New School, UNITED STATES

## Abstract

Our aim was to develop a widely available educational program in which students conducted authentic research that met the expectations of both the scientific and educational communities. This paper describes the development and implementation of a citizen science project based on DNA barcoding of reptile specimens obtained from the Museums Victoria frozen tissue collection. The student program was run by the Gene Technology Access Centre (GTAC) and was delivered as a “one day plus one lesson” format incorporating a one-day wet laboratory workshop followed by a single lesson at school utilising online bioinformatics tools. The project leveraged the complementary resources and expertise of the research and educational partners to generate robust scientific data that could be analysed with confidence, meet the requirements of the Victorian state education curriculum, and provide participating students with an enhanced learning experience. During two 1-week stints in 2013 and 2014, 406 students mentored by 44 postgraduate university students participated in the project. Students worked mainly in pairs to process ~200 tissue samples cut from 53 curated reptile specimens representing 17 species. A total of 27 novel Cytochrome Oxidase subunit 1 (CO1) sequences were ultimately generated for 8 south-east Australian reptile species of the families Scincidae and Agamidae.

## Introduction

A disconnect exists between the way experimental work is conducted in high school science in many developed countries and the way contemporary science is practised in academia and industry [1,2]. The purposes of practical work in science education at secondary school (Grades 7–12) is a perennial topic in education literature (e.g. [3–6]). Educators regard practical work as a means for students to learn technical skills and methodology, crystallise abstract theoretical concepts in a tangible context, and develop “soft skills”, such as collaboration and dialogue, in order to construct meaning and solve problems. School teachers and students value practical work for stimulating students’ interest by creating variety in their learning experiences, as well as promoting opportunities for learning through discovery [7–9].

Scientific research involves investigation to generate, synthesize, and evaluate knowledge, often under circumstances in which the inquiry is open-ended and the methods for generating or analysing the data need to be devised. Scientists accomplish this by building knowledge and experience in a particular field to frame and test hypotheses and to defend their course of investigation. By contrast, practical work in the high school classroom is often prescriptive with predictable results, and most often performed for an illustrative purpose [7]. Students are liable to feel incompetent if they frequently obtain results other than those that are expected, potentially undermining confidence to pursue a science-based career [10,11].

Inquiry-based learning is a contemporary approach to science education. Inquiry-based learning may be structured in different ways, however, it essentially involves students building knowledge by undertaking an authentic investigation, which typically involves some sort of experiment, in order to formulate an answer to a question [12]. Ideally, the process is driven by the students, harnessing their inherent interest in a topic, with the support and guidance of the educator. In reality, however, opportunities for experimental investigations in high school are largely shaped by constraints imposed by the time available, the organisational operation of the school, the technical resources or expertise available in the school laboratory, budgetary restrictions that preclude sophisticated experimentation (such as in molecular biology), and the assessment requirements of the course [3–5,13].

Several educational DNA barcoding projects have emerged as a strategy for school students to engage with and learn about contemporary biology by practising authentic science. DNA barcoding enables species to be identified and classified through sequencing a short region of the genome, the mitochondrial Cytochrome Oxidase subunit 1 (CO1) gene [14]. CO1 is essential for normal cell function and therefore present in all animals. This gene is well suited for DNA barcoding because the rate of spontaneous sequence changes in CO1 is high enough to ensure there are substantial differences between species but low enough to be generally identical within a species. DNA barcoding has many applications, including being used for evolutionary studies, monitoring for biosecurity, ecological surveys, and authenticating the labelling of commercial natural products (e.g. [15–18]). Educational barcoding projects vary in structure, resourcing, the participant age group they target, and in the level of commitment required from students and educators. Some projects give students the opportunity to collect samples in their local environment and submit them for barcoding (e.g. as described by Henter et al [19]). The project coordinators do the wet chemistry and species identifications externally, with students receiving taxon inventories to probe questions about the environment. Other projects are more immersive investigative experiences with students either framing their own investigation with the support of facilitators (e.g. New York Urban Barcoding Project) or apprenticed to lead scientists to work within the framework of existing research projects (e.g. Coastal Marine Biolabs [19–21]). These projects engage the students in the wet chemistry and bioinformatics, which require substantial commitment from the students and their teachers for the necessary training, as well as provision of equipment that is unavailable at the home school.

Inspired by such barcoding projects, we developed an educational barcoding program in Victoria (Australia) that combined authentic science with educational outcomes for senior school students (Years 11–12) who have elected to study Biology. The project was compatible with specific key knowledge points, outcomes, and assessments in Unit 4 of the Victorian Certificate of Education (VCE) Biology study design, titled “Continuity and Change” (Table 1).

**Table 1:** Specific descriptors in the Victorian Certificate Education (VCE) Biology Study Design (2013–16) addressed in the development of the barcoding project.

Key knowledge	Outcomes	Assessment tasks
• Unit 4, Area of Study 1: DNA tools and techniques: gel electrophoresis; DNA amplification; DNA sequencing • Unit 4, Area of Study 2: Evidence for biological evolution over time: molecular homology • Unit 4, Area of Study 2: Determination of evolutionary relationships: comparison of DNA sequences; mitochondrial DNA; phylogeny	• Unit 4, Outcome 1: Analyse evidence for the molecular basis of heredity, and patterns of inheritance • Unit 4, Outcome 2: Analyse and evaluate evidence for evolutionary change and evolutionary relationships, and describe mechanisms for change including the effect of human intervention on evolutionary processes through selective breeding and applications of biotechnology.	• Unit 4, Outcome 1: An investigation using a DNA tool or manipulation technique • Unit 4, Outcome 2: An oral or written report that demonstrates evolutionary relationships using first- or second-hand data.

The project aimed to meet the objectives of four groups of stakeholders while reciprocally accommodating their sometimes conflicting constraints.

The first stakeholders, and core to the entire project, were the school students. We advocate foremost for productive learning experiences that give students a sense of personal satisfaction, growth, and self-esteem [11]. A utilitarian motive, however, shapes the VCE learning experience. Students studying VCE courses aim to complete their high schooling and, most often, prepare for entry into higher education courses. Destination data for Victorian school leavers from 2012 to 2016 (http://www.education.vic.gov.au/ontrack) show that 52.0–54.2% of students completing VCE commenced a Bachelor degree at university the following year. A further 14.6–17.5% started a Diploma course. The objective for the majority of VCE students was academic achievement. Students seek to build an understanding of the concepts contained within the course and to be able to apply those understandings in novel situations. The merits of formal assessment will not be debated here, however, VCE Biology students demonstrated their understanding through assessment of written practical work and an external examination.

The second group of stakeholders were VCE students’ home school teachers. Their objective was to advance the academic success of all their students but they were burdened to manage additional and sometimes conflicting agendas. These included tackling the challenge of student engagement; furnishing formative assessment for progressing student outcomes and summative assessment for reporting to state authorities; meeting the school’s motives of endeavour, competency, and prestige; and–not least–conforming to the restrictive timeframes imposed on preparing and teaching the course.

The third stakeholder was government. The government’s aim is to enhance the quality of education for all students, both for the personal development and literacy of each of its citizens and for the technological and economic progress of the state (e.g. see https://www.ed.gov/stem for USA, http://www.scseec.edu.au for Australia, http://www.education.vic.gov.au/about/programs/learningdev/vicstem and [22] for Victoria). Uniquely in Victoria, six Science and Maths Specialist Centres have been established by the state government as an instrument for delivering high quality science and mathematics education to all Victorian students (http://www.education.vic.gov.au/about/programs/learningdev/vicstem/Pages/centres.aspx). The centres’ programs are crafted through a fusion of contemporary forms of pedagogical practice, scientific expertise, and technology [23,24]. The principal remit of the six centres is equitable student and teacher access to high quality science and mathematics education. The centres are obligated to host approximately half their programs for students from government schools located in rural or low socioeconomic metropolitan localities.

The first three groups of stakeholders were essentially nested within one another: government through its education ministry, schools and their teachers, and the individual students. The fourth group, the scientists, were superficially independent. From an academic viewpoint, scientists are focussed on producing, evaluating, and communicating data to develop or refine scientific models. From an applied viewpoint, scientists are concerned with building knowledge that develops, informs, or reforms societal, economic, or technological objectives [25]. In a competitive funding environment, scientists are compelled to demonstrate value for money spent on their projects. Whether internally or externally funded, time and resources are therefore primarily dedicated to delivering research outcomes. Depending upon the researcher’s position, they may also be absorbed by commitments to delivering undergraduate courses, postgraduate training, managing a laboratory or research program, and other administrative duties. The ideal of public outreach appeals to many scientists but the reality is that there tends to be little opportunity in their hectic schedules to accommodate it. There are, however, complementary benefits to scientists and the public when scientists become involved in public outreach [26–28]. These include enhancing scientists’ communication skills; raising the profile of the scientist’s research in the community, which may inform publicly funded projects; meeting government objectives of enhancing the quality of education; and cultivating student interest in science.

We structured the DNA barcoding project as a citizen science educational program. The program was delivered as a “one day plus one lesson” format. The wet chemistry was carried out by students during a one day educational workshop in a specialist facility, the Gene Technology Access Centre (GTAC). The bioinformatics analysis of the data was subsequently done in a single lesson at the students’ home school. Our educational barcoding format allowed scientists and educators to collaborate flexibly to provide students of upper high school years with an authentic scientific experience. In our format, students worked directly with scientist mentors in small groups to enhance the scientific experience and facilitate learning. The data generated were accessible to all participating students so they had the latitude to pursue deeper inquiry.

Vertebrate fauna are inherently interesting and engaging to school students, as well as being of scientific importance. We used Victorian native reptiles as the subject of this DNA barcoding program. Sequence libraries of the mitochondrial gene CO1 have been highly effective as a DNA barcode to identify and distinguish species of herpetofauna in Africa, Asia, Europe, North America, and New Zealand [29–34]. Despite the enormous diversity of reptiles in Australia, sequences of Australian fauna are currently relatively underrepresented in databases. The specimens DNA barcoded in this project were collected during “Bioscans”, field trips in which Museums Victoria scientists collect and record a snapshot of the biodiversity of a region within targeted habitats. These Bioscans were conducted in Victoria’s Alpine National Park in the east of Victoria and the Grampians National Park in the west of the state. Previous studies of reptiles, as well as invertebrate fauna, indicate that eastern Australian highlands sustain endemic species that have diversified locally in habitat “islands” [35,36]. The collection sites were some 500 km apart with major peaks exceeding 1,800 m in the Alpine National Park and 1,100 m in the Grampians.

DNA barcodes provide a resource for advancing biogeographical and evolutionary studies of Victorian fauna. Enhanced understanding of the genetic diversity within species also informs conservation management, particularly in regions that are sensitive to the impacts of climate change. Here we report on proof of concept that a citizen science project based on DNA barcoding provides both valuable educational experiences for school students and beneficial data for the scientific community.

## Materials and methods

### Format and time frame

Program delivery was designed so that the wet laboratory workshop incorporated DNA tools and manipulation techniques relevant to Unit 4, Area of Study 1 of the course (see Table 1). A bioinformatics lesson was designed to analyse the data generated back at school as an application of molecular homology for determining evolutionary relationships. Both the wet laboratory and the bioinformatics tasks met the assessment requirements of course outcomes 1 and 2 respectively. Class groups were booked in by teachers to complement their regular course. The experimental work was carried out by students and assessed as required by their teachers. While the program aligns with the assessment requirements of VCE Biology, each teacher sets authentication questions for their own class to avoid replicating assessments system-wide. The practice is mandated by Victorian Curriculum and Assessment Authority (VCAA) and teacher-generated assessments are audited by VCAA to ensure compliance. The program promoted the ideal that students would be recognised for their contribution to the project and students were given the option to record their names as contributors.

### Educational support

**Fig 1:**
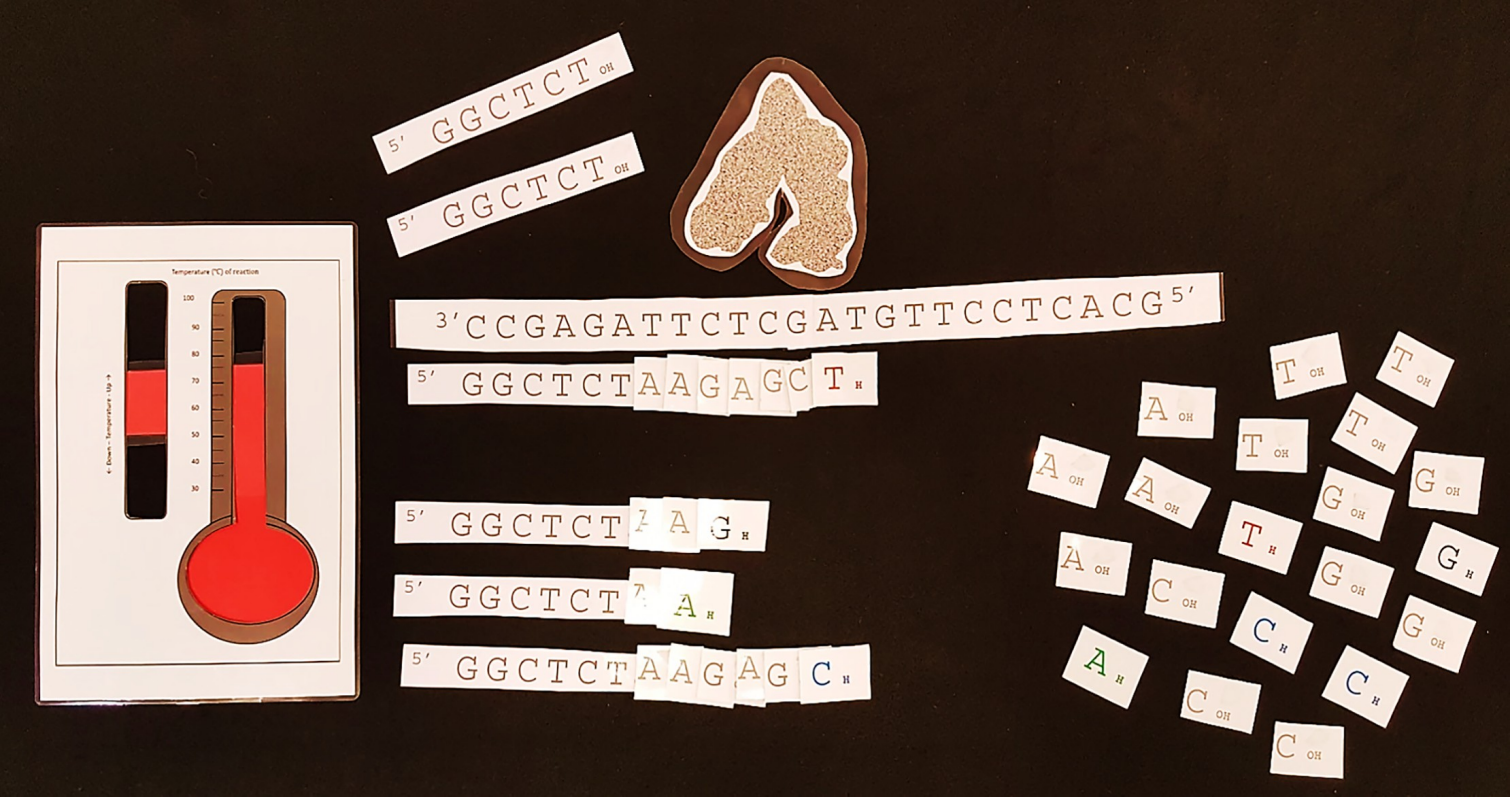
An example of the models used for educational purposes in the DNA barcoding program at GTAC.

**Fig 2:**
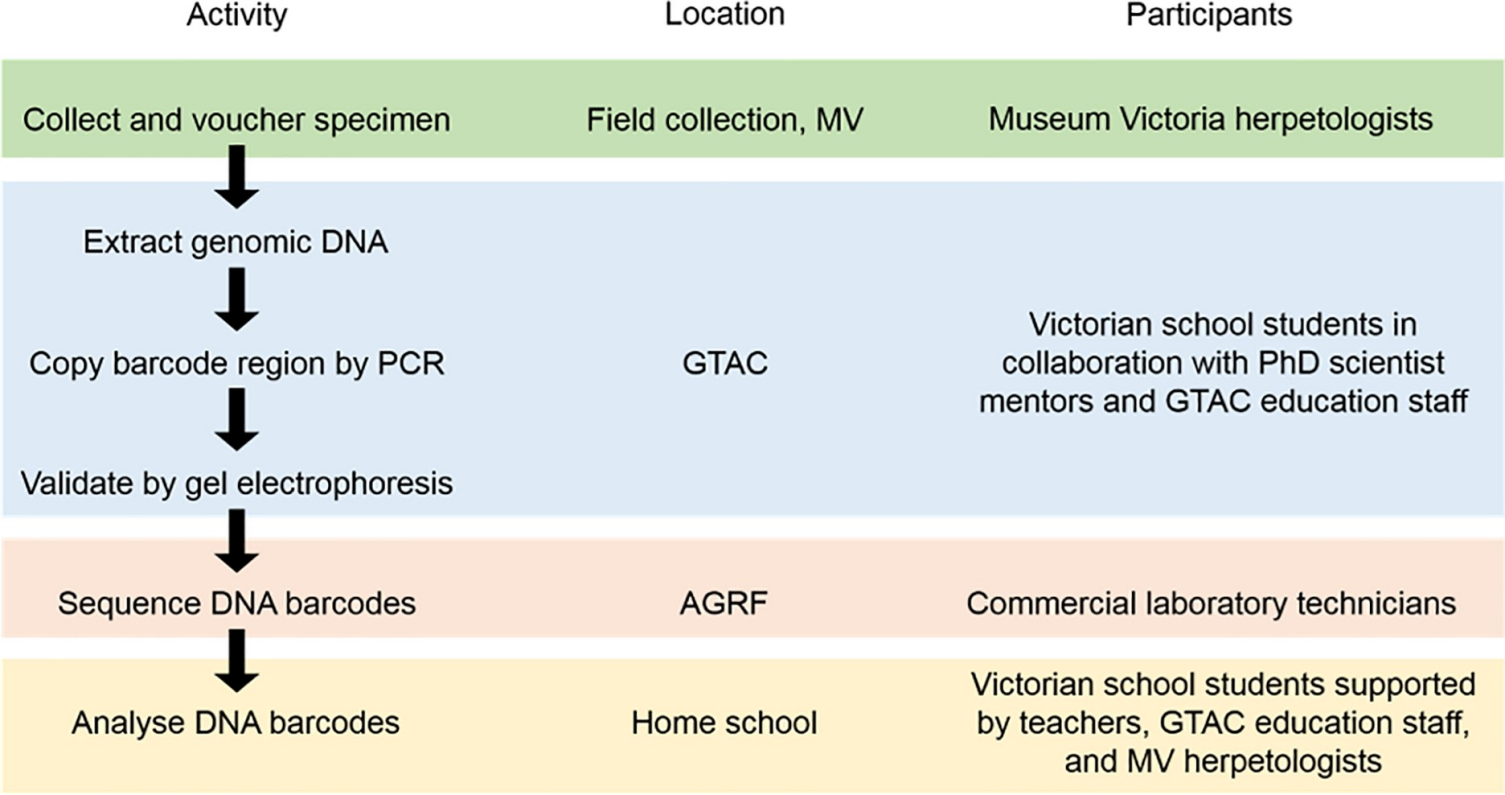
Work flow for the educational DNA barcoding project.

The program was taught with the preliminary assumption that students may be inducted into the project with essentially no understanding of the concept of DNA barcoding or the DNA manipulation techniques involved. The educational program based on the project was crafted according to a social constructivist approach [37,38]. The laboratory work was done by groups of usually six students, collaborating with a PhD scientist mentor. At GTAC, scientist mentors are predominantly postgraduate university students studying for their PhD. Within each group of six, 3 pairs of students each processed a reptile subsample. Students were supported to do the work through discussions with their peers, their scientist mentor, and GTAC educators, as well as through support material in the form of instructional workbooks, explanatory PowerPoint animations, and physical models that were developed by GTAC educators. An example of models used for the program is shown in Fig 1. The workflow to accommodate a day program is outlined in Fig 2.

The model comprises laminated pieces including thermometer, Taq enzyme, target DNA sequences, primers, individual dNTPs and individual coloured ddNTPS (black G, green A, red T, and blue C). Attached to the dNTPS are hooked Velcro on the upper 3’ end and looped Velcro on the lower 5’ end. Primers have only the hooked Velcro at the 3’ end and ddNTPs have only looped Velcro at the 5’ end. Students manipulate the pieces to learn and demonstrate their understanding about the PCR process and Sanger sequencing reactions.

The work completed by school students at GTAC comprised the one-day wet lab program. The DNA barcodes are analysed by students during a lesson at the home school.

Students were invited to complete a feedback survey at the end of the wet laboratory workshop to evaluate the learner experience. Students were asked to rate five statements on a 5-point scoring system according to the extent to which they agreed or disagreed with the statements. The surveys were conducted anonymously and voluntarily, took less than five minutes to complete, and the data were aggregated and reported as the average score for all student responses to each feedback statement.

### Specimen collection and sampling

No experiments were conducted on living animals, nor were animals collected specifically for this project. Liver samples used for DNA barcoding were subsampled from museum specimens that were already stored in the frozen tissue collection at Museums Victoria. Specimens were collected during Museums Victoria’s annual field surveys (referred to as “Bioscans”) and the collection data are listed in S1 Table. The material was loaned to the project following approval under MV’s research tissue grant transaction #DNA-2013-5 and #DNA-2014-11. Ultimately, the project included 53 tissue samples representing 17 species of the Australian reptile families: Scincidae, Agamidae, and Gekkonidae, in the order Squamata. Four tissue samples from two south eastern Australian frog species were also trialled for the project (S1 Table).

Multiple subsamples were dissected from each liver specimen. Following excision, subsamples were stored in 95% ethanol until processed by students in the wet laboratory workshops.

### Implementation of wet laboratory workshops

Wet laboratory methods were undertaken by participating Unit 4 VCE Biology students in a single day at GTAC (Fig 2). The workshop and accompanying training fit within a standard school day and incorporated DNA extraction, PCR, and validation of PCR product by gel electrophoresis.

Students adopted a subsample of liver tissue that had been prepared for them in advance for the workshop. Total genomic DNA was extracted using the Nucleospin Tissue Kit (Machery-Nagel, Germany) following the manufacturer’s protocols.

The mitochondrial CO1 gene was targeted with primers developed by Nagy et al [29] (forward primer, 5'-TNT TMT CAA CNA ACC ACA AAG A-3' and reverse primer, 5'-ACT TCT GGR TGK CCA AAR AAT CA-3') at concentrations of 1 μM each, yielding a PCR fragment of up to 664 base pairs in length. The CO1 gene segment was copied from the purified genomic DNA using the Phusion HotStart Taq DNA polymerase (New England Biolabs, USA) with accompanying reagents according to the manufacturer’s protocols. The PCR temperature cycle on the BioRad model T100 thermocyclers incorporated denaturation at 98°C for 30 s, annealing at 48°C for 30 s, and primer extension at 72°C for 45 s. The cycle was repeated 25–30 times and bracketed by an initial denaturation step at 98°C for 2 min and a concluding extension step at 72°C for 5 min. PCR products were evaluated by 1% agarose gel electrophoresis stained by SYBRSafe (Invitrogen, USA) and compared against size standards in Quick Load PCR Marker #N0475S (New England Biolabs, USA).

After the student workshops were completed, PCR products were purified using the QIAquick PCR Purification Kit (Qiagen, Germany) following the manufacturer’s protocol. Purified PCR products were submitted to the Australian Genome Research Facility (AGRF) for bidirectional sequencing using the same primers and at the same concentrations as those used for PCR. Concentrations of template DNA for sequencing reactions were adjusted according to their staining intensity by 1% agarose gel electrophoresis and verified empirically to be in the range 15–30 ng/μL using the NanoDrop ND-1000 spectrophotometer (Thermo Fisher Scientific, UK).

### Data processing

The ab1 files containing raw sequence data were uploaded and processed by GTAC staff in the DNA Subway website (https://dnasubway.cyverse.org/). Raw sequences were trimmed and the consensus sequences derived from forward and complementary reverse sequencing reactions. Processed CO1 sequences containing 664 nucleotides each were uploaded for analysis by students in the Biology Workbench bioinformatics platform (http://workbench.sdsc.edu/). Sequence alignments were generated using CLUSTALW [39,40] and unrooted phylogenetic trees drawn using PHYLIP [41] available at the website. Unrooted phylogenetic trees were generated following the methods described in [42] model using the MEGA7 software package [43].

To validate specimen sequences, subsamples cut from each specimen were processed independently by different students from different schools working with different scientist mentors on different days and in different locations. Specimen sequences were considered validated if sequences generated from different subsamples were identical. Generated sequences were evaluated further by comparison with published sequences of the same gene region from other reptile species. Sequences were downloaded from Genbank, aligned with the new sequences using ClustalW and analysed using free sequence analysis software Mega7 (http://www.megasoftware.net/). Jukes-Cantor genetic distance measurements were calculated within each species clade and among all species pairs, and a phylogenetic tree was assembled with maximum likelihood methods using the basic settings.

## Results

During two 1-week stints in 2013 and 2014, 406 students and 44 scientist mentors participated in the DNA barcoding program. They collectively processed 57 specimens representing 19 species of south eastern Australian reptiles and amphibians. In the students’ hands, up to 44% of PCR reactions yielded product at the first PCR attempt. PCR products were ultimately generated and sequenced for 28 specimens representing 27 individuals from 8 reptile species of the families Scincidae and Agamidae. Of the specimens examined, 25 met our criteria for validated sequences, with single subsamples yielding sequences for the other three (Z29155 and Z27172, both Jacky dragons, and Z27192, a Cunningham’s skink). All 27 sequences were uploaded to GenBank (Table 2). Tissue specimens from two frog species were trialled (S1 Table), however, these yielded no PCR products after repeated attempts and were not investigated further.

**Table 2:** Summary of specimens that yielded sequences that were deposited in Genbank.

Common name	Scientific name	MV Rego #	Voucher specimen	Collection date	Locality	Genbank accession number
Jacky dragon	*Amphibolurus muricatus*	**Z27172**	D75634	Nov-13	Alpine National Park, Cobberas track	MH028635
		**Z29155**	D75668	Nov-13	Alpine National Park, Beloka Road Granite Outcrop	MH028622
Copper tailed skink	*Ctenotus taeniolatus*	**Z29307**	D75731	Nov-13	Alpine National Park, Snowy River Road	MH028632
Cunningham's skink	*Egernia cunninghami*	**Z27192**	D75654	Nov-13	Alpine National Park, Beloka Road Granite Outcrop	MH028630
Black rock skink	*Egernia saxatilis intermedia*	**Z26887**	D75751	Nov-13	Alpine National Park, Ramshorn peak to carpark and moth cave	MH028629
		**Z26891**	D75754	Nov-13	Alpine National Park, Ramshorn peak to carpark and moth cave	MH028609
		**Z29306**	D75730	Nov-13	Alpine National Park, Limestone Road, waterfall	MH028628
		**Z27240**	D75700	Nov-13	Alpine National Park, Rams Horn	MH028617
Southern water skink	*Eulamprus tympanum tympanum*	**Z26840**	D75592	Nov-13	Alpine National Park, road to Rams Horn 3	MH028627
		**Z27151**	D75596	Nov-13	Alpine National Park, Native Dog Flat	MH028633
		**Z27153**	D75598	Nov-13	Alpine National Park, Rocky Plain Creek, off Limestone Creek	MH028623
		**Z27158**	D75603	Nov-13	Alpine National Park, creek crossing below Davies Plain Hut Campground	MH028619
		**Z29168**	D75723	Nov-13	Alpine National Park, Limestone Road, Bouyard Creek crossing	MH028618
Grass skink	*Lampropholis guichenoti*	**Z27144**	D75585	Nov-13	Alpine National Park, Limestone Road, Native Dog Flat Campground	MH028610
		**Z29163**	D75719	Nov-13	Alpine National Park, Bulley Creek at Cowombat Track	MH028634
		**Z29165**	D75725	Nov-13	Alpine National Park, Rocky outcrop on Cowombat Track	MH028611
		**Z29305**	D75729	Nov-13	Alpine National Park, Limestone Road, waterfall	MH028624
		**Z29309**	D75734	Nov-13	Alpine National Park, McFarlane Flat Track, Berrima Creek crossing	MH028620
Tussock skink	*Pseudemoia entrecasteauxii*	**Z27130**	D75888	Nov-13	Alpine National Park, road to Rams Horn 3	MH028613
		**Z27145**	D75586	Nov-13	Alpine National Park, Rams Horn Track	MH028621
		**Z27154**	D75599	Nov-13	Alpine National Park, Rocky Plain Creek	MH028625
		**Z27182**	D75644	Nov-13	Alpine National Park, Limestone Road, Native Dog Flat Campground	MH028612
		**Z27232**	D75696	Nov-13	Alpine National Park, Davies Plain track	MH028631
Mountain dragon	*Rankinia diemensis*	**Z27139**	D75897	19/11/2013	Victoria, Alpine National Park, Rams Horn Track	MH028615
		**Z27169**	D75631	Nov-13	Alpine National Park, road to Rams Horn 3	MH028614
		**Z27171**	D75633	Nov-13	Alpine National Park, Cowombat track near Murray River	MH028616
		**Z27183**	D75645	Nov-13	Alpine National Park, Cowombat Flat Track	MH028626

A total of 664 nucleotides of the CO1 gene were examined. Of these, 51.7% were conserved across the data set of 27 reptile sequences. Table 3 summarises the number of nucleotide differences between reptile specimens of the same nominal species and between members of other nominal species. This type of analysis is amenable to secondary school students and is patently transparent as the data can be gathered directly from sequence alignments. The number of nucleotide differences among members of each species ranged from no difference between two specimens of *Amphibolurus muricatus* (Jacky dragon) to 28 nucleotide differences between specimens of *Egernia saxatilis ssp*. *intermedia* (black rock skink). Sequences were generated for four black rock skinks as the liver of one animal (D75751) was cut and curated as two separate specimens in the tissue bank (Z26887and Z26888, S1 Table). Among them, three black rock skinks had identical sequences and the sequence of the fourth animal accounted for all 28 nucleotide differences.

**Table 3:** Sequence divergences within and between nominal species.

No. sequence		Copper tailed skink	Cunningham's skink	Black rock skink	Southern water skink	Grass skink	Tussock skink	Mountain dragon	Jacky dragon	Snake-eye skink	Sand lizard
1	Copper tailed skink	– / –	0.258	0.248	0.267	0.271	0.284	0.442	0.435	0.258	0.28
1	Cunningham's skink	**142**	– / –	0.132	0.286	0.261	0.258	0.407	0.399	0.227	0.273
4	Black rock skink	**149**	**92**	**28** / 0.018	0.269	0.263	0.233	0.422	0.419	0.229	0.26
5	Southern water skink	**152**	**163**	**168**	**13** / 0.009	0.322	0.288	0.431	0.432	0.286	0.256
5	Grass skink	**171**	**162**	**175**	**194**	**45** / 0.057	0.228	0.391	0.39	0.235	0.272
5	Tussock skink	**162**	**158**	**155**	**167**	**163**	**26** / 0.018	0.434	0.396	0.22	0.26
4	Mountain dragon	**220**	**211**	**224**	**226**	**221**	**228**	**12** / 0.01	0.147	0.412	0.38
2	Jacky dragon	**218**	**205**	**223**	**219**	**278**	**213**	**93**	**0** / 0	0.391	0.365

The lower left triangle (bold text) represents the number of nucleotide differences between 664 nucleotides of the mitochondrial CO1 gene. The upper right triangle (plain text) represents the number of base substitutions per site from averaging over all within-species pairs (using 25 sequences) and between-species pairs (using 31 sequences) according to the Jukes and Cantor [44] model. All positions containing gaps and missing data were eliminated, with a total of 633 positions in the final datasets. Evolutionary analyses were conducted in MEGA7 [43]. The dash symbol “–”in the results denotes cases in which it was not possible to estimate evolutionary distances.

The number of nucleotide differences between nominal species ranged from 92 nucleotides between representatives of *Egernia saxatilis ssp*. *intermedia* (black rock skink) and *E*. *cunninghami* (Cunningham’s skink) to 278 nucleotides between representatives of *Amphibolurus muricatus* (Jacky dragon) and *Lampropholis guichenoti* (grass skink). Sequences for the two representative species of the family Agamidae (*Amphibolurus muricatus*, Jacky dragon and *Rankinia diemensis*, mountain dragon) differ by 93 nucleotides. Sequences for the Agamidae species differ by 211–278 nucleotides from those of the other six species, all representatives of the family Scincidae. Within the family Scincidae, sequences vary by 92–194 nucleotides.

The observations from nucleotide difference data were corroborated by Jukes-Cantor statistical measures of evolutionary divergence within and between nominal species (Table 3). Three additional CO1 sequences from reptiles were downloaded from Genbank and added to the dataset to evaluate the sequences obtained in the student project. These were JN871612.1 the sand lizard (*Lacerta agilis*); KC349619.1 grass skink (*Lampropholis guichenoti*); and KF604772 snake-eyed skink (*Cryptoblepharus boutonii*).

**Fig 3:**
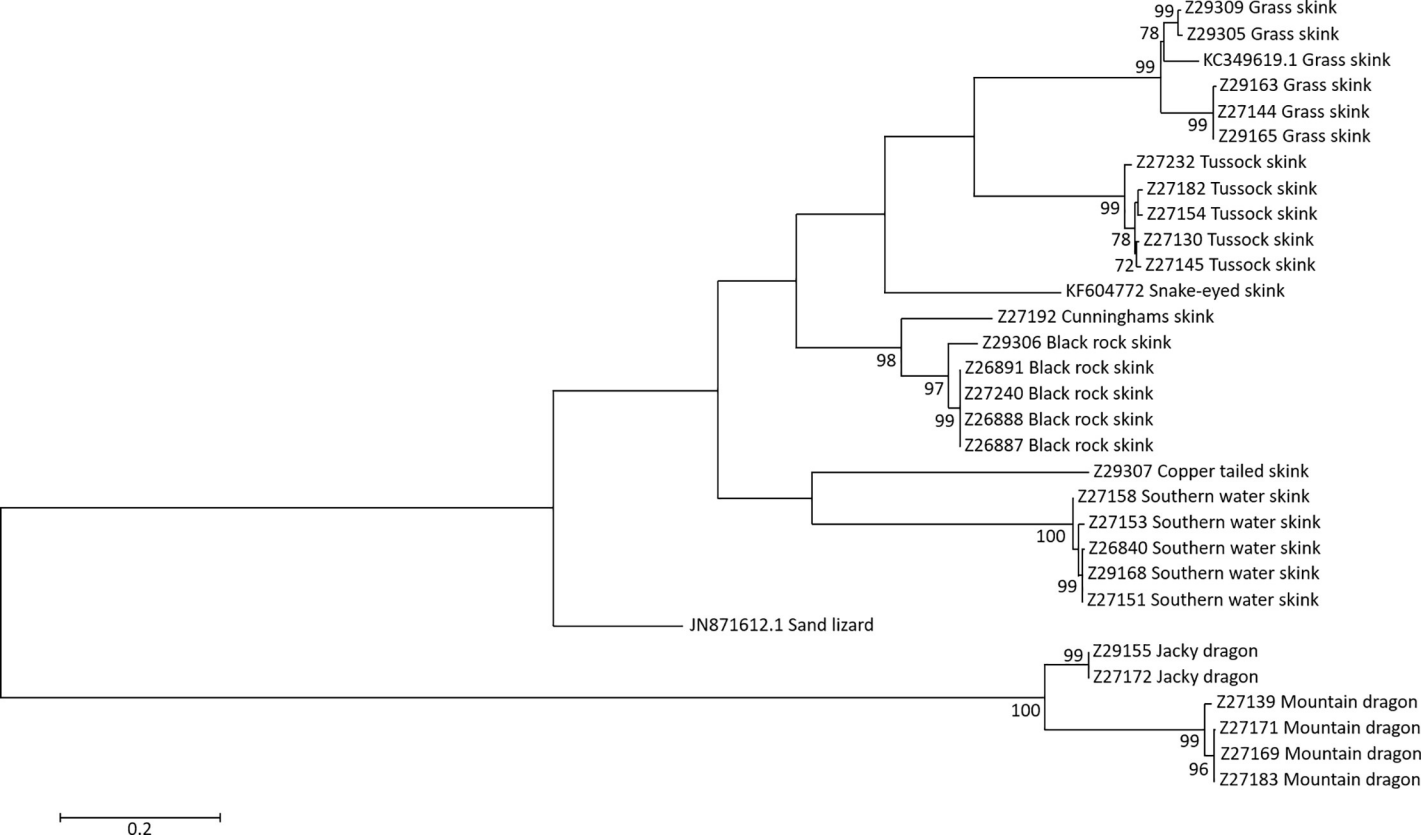
A phylogenetic tree depicting relatedness between 31 mitochondrial CO1 sequences representing 9 species of Victorian skinks.

A phylogenetic tree for the complete data set (including the three published CO1 sequences) was generated using a Maximum Likelihood analysis with a Hasegawa-Kishino-Yano model; the tree with the highest log likelihood is shown in Fig 3, with bootstrap values inferred from 2000 replicates. The sequences for the two Agamidae species cluster on a distinct and well supported branch of the tree. The tree highlights two distinct and well supported clades within the grass skinks clusters, as well as within the black rock skink clade. The remaining Scincidae form discrete, relatively evenly spaced clusters corresponding to nominal species belonging to separate genera.

The sequences were aligned using ClustalW available from the SDSC Biology Workbench and phylogenetic analyses were conducted in MEGA7 [43]. The tree was inferred using the Maximum Likelihood method based on the Hasegawa-Kishino-Yano model, with bootstrap values inferred from 2000 replicates. Only bootstrap values greater than 70% are shown on branches. The tree is drawn to scale, with branch lengths measured in the number of substitutions per site. The analysis involved 31 nucleotide sequences, 28 generated in this study and 3 downloaded from Genbank. There were a total of 644 positions in the final dataset.

**Fig 4:**
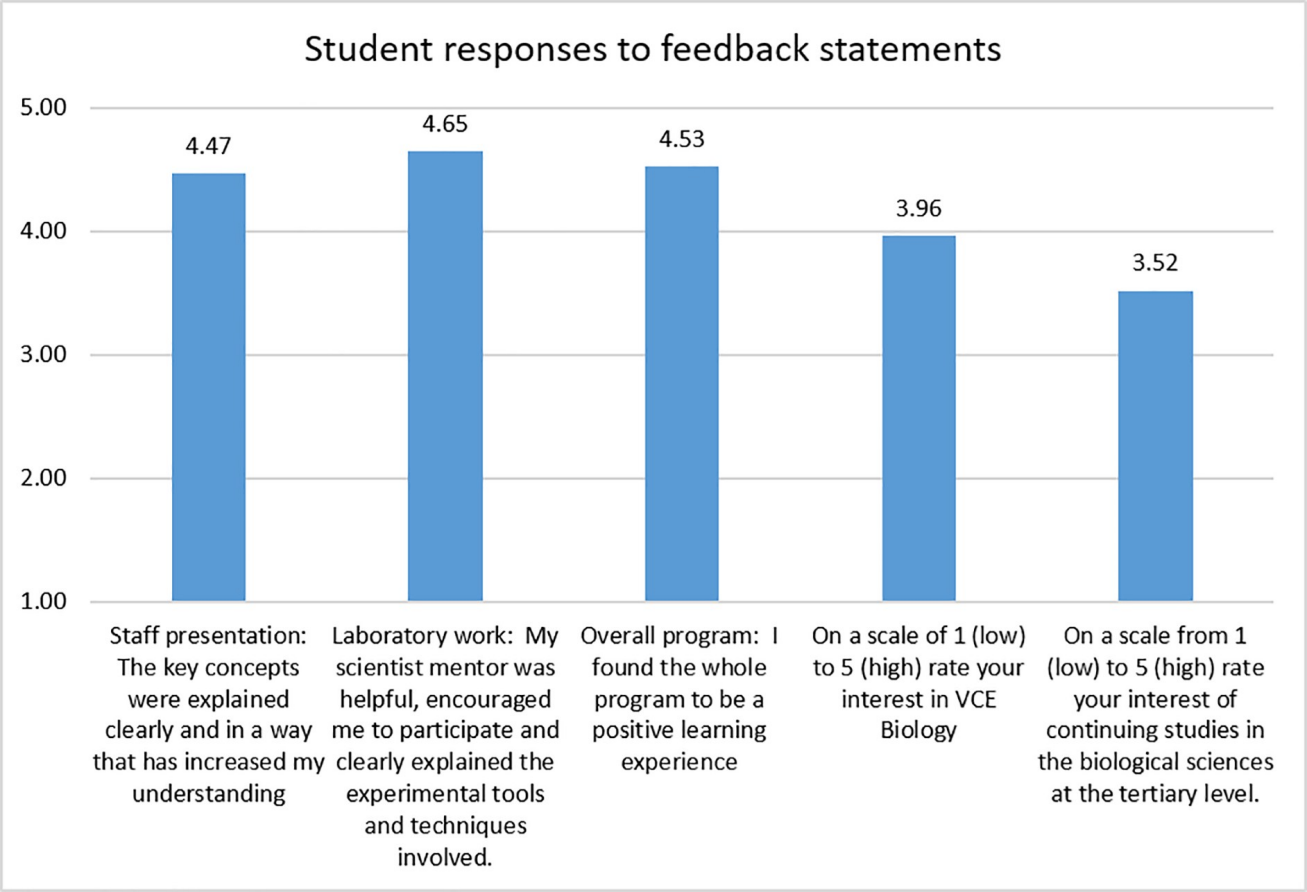
Summary of student feedback given at the conclusion of the educational wet laboratory program.

Student responses to a feedback survey at the conclusion of the DNA barcoding program are presented in Fig 4. Participating students gave an average score above 4.4 out of 5 for positive statements about the presentation by the GTAC education officer, the wet laboratory workshop with the PhD scientist mentor, and the overall experience of the program. These average scores exceeded those students gave for their interest in the school subject or their interest in further study.

Students were asked to score feedback statements using the following criteria: 1 (strongly disagree), 2 (disagree), 3 (neither agree nor disagree), 4 (agree), or 5 (strongly agree). Data represent average scores for responses provided by 398 students participating in the program during 2013 and 2014.

## Discussion

The project outlined here represents a collaboration that we believe is an effective model for connecting scientists with educators and school students to deliver an enhanced learning experience. The design of the research project and the educational program was informed by the complementary expertise of both research scientists and the GTAC educators. Collaboration involved developing, trouble shooting, adapting, and scaffolding the experimental protocols so that they were suitable for students. The educational program was delivered by GTAC educators together with PhD scientists. The hallmark of the educational program is school students utilising contemporary biotechnology to carry out authentic research in close collaboration with their scientist mentors.

It has long been recognised that summative assessment compels teachers to select the type of practical work students do, particularly at senior levels of school science [4,5]. A demonstrative example is the VCE Biology study design that was implemented until 2016. Between 2013 and 2016, enrolments in VCE Biology Units 3 and 4 grew from 12,364 to 13,661 per year (VCAA: Joe Bui, pers. comm). Anecdotally, the course was regarded as stressful by both students and teachers because it was content-heavy, externally examined, and there was limited time or opportunities for practical work. The practical work that was done was largely conducted with the aim of fulfilling the assessment guidelines of the course. In Units 3 and 4 of the course (Year 12 equivalent), students were required to submit ten pieces of work as school-assessed coursework, at least six of which were mandated as reports on specific practical investigations (VCAA VCE Biology study design, http://www.vcaa.vic.edu.au/documents/vce/biology/biologysd-2013.pdf). Normalising assessment within class cohorts further constrained students to follow prescribed practical work and, in our experience, students were often preoccupied with getting the “right” answer to meet their assessment requirements.

For these reasons we sought to engage VCE Biology students in practical work that offered the prospect of generating authentic scientific data without compromising the assessment requirements or schedules imposed by the course. DNA barcoding presented a viable instrument to achieve these ends. It is a contemporary theme in life science with many potential research and commercial applications [45] and, as with other educational barcoding programs, the modular workflow of DNA barcoding allowed the educational program to be tailored to address specific curriculum objectives [19]. The barcoding format we developed comprises a one-day wet laboratory workshop plus a single bioinformatics session that is conducted online (Fig 2). In line with curriculum requirements, the workshop comprised DNA extraction, PCR, validation of PCR products by gel electrophoresis and an introduction to Sanger sequencing. The educational program provided training for participants, was streamlined to fit within school structures, and delivers the educational outcomes of the VCE Biology course (Table 1).

For this educational barcoding format, specimen collection and vouchering was conducted by Museums Victoria scientists. This achieves several ends. First, it legitimises the practical work by anchoring it to a genuine scientific enterprise [46,47]. It raises the stakes because students appreciate they are not replicating a “cook-book” practical but are taking a very real risk to generate data that are new to science. In return, the students’ work is recognised and valued by the scientific community. Students participating in educational barcoding projects consequently report increasing pride, confidence, and scientific identity for their endeavour [19]. Second, to make the project scientifically credible, the vouchers must be preserved for the long term but such repositories are beyond the capacity of most schools. Specimen collection, management, and the associated legal regulation fall within the domain of research institutions, such as museums, making them the most appropriate custodians of barcoding vouchers, even if the public is enlisted to undertake the species identification [45]. We recognise that any form of data collection is excellent practice for school students, however, this kind of activity was not directly assessable in the VCE Biology study design so it was one aspect of barcoding that was omitted from the student program.

The wet laboratory workshops were delivered in a student-centred social constructivist environment [37,38] in which students interacted with their peers, their PhD scientist mentor, and GTAC educators. By working in small groups, every student is encouraged to participate, and provides a relational environment that is conducive to learning [11]. The workshop was crafted with the assumption that students had no knowledge of DNA barcoding. In practice, participating students had different levels of prior knowledge and sometimes misconceptions about the techniques or the underlying theoretical aspects. In traditional school settings, students doing practical work do not necessarily have opportunities to draw links between observation and theory [7]. In the wet laboratory workshops for our project, pedagogical aids such as animations and physical models were used to support student discussions. PowerPoint animations designed by GTAC educators helped students to visualise molecular concepts. The pedagogical aids were delivered on a “need-to-know” basis within the experimental protocols [48] so that they contributed to a coherently structured educational program. The physical models enabled students to work in groups manipulating props to visualise and explore complex processes, demonstrate their understanding, and interrogate their own and each other’s understandings [49]. Student interactions using these pedagogical aids enabled GTAC educators and PhD scientist mentors to formatively assess student understanding.

The student experience was core to the program. Feedback indicated students regarded the workshop as a positive learning experience, and the interaction with their PhD scientist mentor was the most valued aspect of the program. Written student comments indicated various reasons for this. First, students identified the value of the educational support PhD scientist mentors offered. At GTAC, educators coach the PhD scientists in pedagogical techniques, such as dialogic discourse [50,51], to help the mentors guide student conversation. This has the reciprocal benefit of training young scientists in communication skills to engage with a public audience [26,28]. Second, the PhD scientist mentors expose students to the diversity of research areas possible in science. The mentors informally discussed their own research and lifestyle with the students, providing the students with valuable insights into a career in science. Third, interactions with PhD scientist mentors dispel students’ perceptions about scientist stereotypes [52,53]. The group of PhD scientists involved in this project were male and female, relatively young (mostly under 30 years of age), and from diverse ethnic and cultural backgrounds. For many students, the PhD scientist mentor is their first extensive contact with a practising scientist, and most came away from the experience warmly regarding their mentors as approachable and amiable. In essence, PhD scientist mentors are role models for the students, and working closely with a PhD scientist mentor builds students’ confidence to engage with science personally and professionally. The scientist mentors’ influence especially benefits students who are marginalised from considering science careers because of scientist stereotypes [54].

Our model has the characteristics of curriculum-based citizen science [55]. It overcomes many of the hurdles identified in translating citizen science to a formal educational setting [13,19,55,56] by aligning the project with the mandated study design and centralising the project to address the challenges of time limitations, involuntary student participation, and the tension between educational and scientific goals. In our model, executive oversight of the research project lay with Museums Victoria and GTAC but participating students were outfitted with the resources and fundamental training required to contribute their own data. The wet laboratory workshops were hosted by GTAC, with students collaborating in small groups with PhD scientist mentors. These arrangements obviate the need for home school teachers to research, develop, and implement the practical work on their own while providing their students with the technology and a social constructivist environment that promotes 21^st^ century competencies [49]. The full-day format for the workshops allowed schools to visit as an excursion that fit within the confines of a regular school day (approximately 9.30 am to 3.00 pm). This centralised structure made the program available to a large number of students from a wide geographical region while standardising and supervising the protocols to ensure they were applied consistently.

Adjusting aspects of the protocol is likely to improve technical outcomes and the student experience. For example, the success rate for PCR reactions increased when students were provided with solutions containing the primers and the Phusion Hot Start cocktail. This reduced the effects of pipetting errors that may have occurred when setting up the PCR reaction. Even so, specimens of some species consistently yielded PCR product, whereas specimens of others consistently did not. Higher rates of successful PCR reactions may therefore be achieved in future by optimising primer design and appropriate sample selection.

The reliability of the data was validated by different students processing different subsamples of the same specimens and generating identical sequences for the subsamples in almost all cases. The veracity of the students’ sequences was further appraised by comparison with published reptile CO1 sequences in Genbank. A bootstrapped phylogenetic tree produced using the new sequences and the three published CO1 sequences show that all sequences cluster together (Fig 3). A published sequence of the grass skink CO1 clusters with the sequences generated for grass skinks in this study. The published snake-eyed skink sequence forms a well-supported clade with the other skink species and the sand lizard, which is from a different family to either skinks or dragon lizards, falls outside both groups. High bootstrap support was found for sequences within each species, but low support for deeper branches is as expected due to the distant relationships amongst the species sampled.

A couple of insights provided by the data demonstrate how citizen science assists researchers. For example, initial analysis of the data revealed a deep split within the grass skink and the black rock skink clades. To rule out the possibility that the specimens were misidentified in the field, Museum Victoria researchers first checked the morphological identification of these individuals using the preserved specimens from which each tissue sample was taken. Morphological identifications appeared to be correct, suggesting that the deep splits found in each clade in this project were a result of cryptic genetic diversity within each species. It’s uncertain why this degree of divergence occurs but a substantial genetic break was also found in populations of White’s skink (*Liophilis whitii*, as *Egernia whitii*) in the Victoria Alps [35]. Our data have stimulated Museums Victoria staff to further research the genetic diversity of the black rock skink in the Victorian Alps.

The sequences for this project have been uploaded to GenBank (Table 2), and there is scope to migrate the data to the Barcode of Life Database (BOLD, cf. [20]). There are currently only 302 sequences of Australian reptiles in the BOLD database so the 27 new CO1 sequences in this study would be a valuable addition.

Our citizen science model also enables inquiry-based learning for students ([12]. The model standardises the wet laboratory method used to generate the data, however, data analysis is guided by the individual interests of the participants, as well as the needs of the project. For example, depending upon the specific research questions they frame, students can scrutinise the entire student data set, or investigate how their contributed data relates to specific subgroups of the complete data set, or study a single nominal species of interest to them, or develop more sophisticated investigations by augmenting the student data with sequences from GenBank. We uploaded the processed barcode sequences to the Biology Workbench website and provided teachers and students with instructional materials for analysing the complete data set. Biology Workbench became unavailable at the end of 2017, however, alternative online bioinformatics platforms, such as GenomeNet (http://www.genome.jp/), are suitable for student use. Following up with teachers, our experience was that uptake of the data analysis component was patchy, ranging from those schools in which students analysed the complete data set according to the instructional materials provided to those schools in which students did no data analysis. Anecdotally, teachers that did not pursue the data analysis indicated it was because the wet laboratory was sufficient to meet the needs of their assessment or the teacher lacked confidence with the bioinformatics. The latter highlights the requirement for targeted teacher training, which can conceivably be embedded within the structure of the one-day workshop when the school group visits GTAC.

The VCE Biology study design was revised for implementation during 2015–2021 (http://www.vcaa.vic.edu.au/Documents/vce/biology/BiologySD-2016.pdf). The updated study design omits DNA sequencing but now explicitly lists bioinformatics and applications of mitochondrial DNA while retaining PCR, gel electrophoresis, and molecular homology, all of which are concepts that can be addressed with DNA barcoding. The new study design also incorporates an extended investigation component, with prospects for students to use barcoding for user-defined investigations. Bioinformatics and biotechnology, including the use of enzymes to manipulate DNA, gel electrophoresis, and PCR, feature in the Australian senior biology curriculum (https://www.australiancurriculum.edu.au/senior-secondary-curriculum/science/biology/). These curriculum developments favour opportunities to adapt educational barcoding to senior high school Biology throughout Australia, providing students with an authentic research-based practical program aligned with their curriculum.

## Supporting information

S1 Table Collection data for specimens investigated in the project with attributions to contributing students and PhD scientist mentors. (PDF)

## References

[1] Haig M., France B, Forret M. Is ‘doing science’ in New Zealand classrooms an expression of scientific inquiry? Int J Sci Educ. 2005;27: 215–226.

[2] Hume A, Coll R. Student experiences of carrying out a practical investigation under direction. Int J Sci Educ. 2008;30: 1201–1228.

[3] Hofstein A, Lunetta VN. The laboratory in science education: Foundations for the twenty-first century. Sci Educ. 2004;88: 28–54.

[4] Abrahams I, Saglam M. A study of teacher’s views on practical work in secondary schools in England and Wales. Int J Sci Educ. 2010;32: 753–768.

[5] Abrahams I, Reiss MJ, Sharpe RM. The assessment of practical work in school science. Stud Sci Educ. 2013;49: 209–251.

[6] Martindill D, Wilson E. Rhetoric or reality? A case study into how, if at all, practical work supports learning in the classroom. Int J Lesson Learn Stud. 2015;4: 39–55.

[7] Abrahams I, Millar R. Does practical work really work? A study of the effectiveness of practical work as a teaching learning method in school science. Int J Sci Educ. 2008;30: 1945–1969.

[8] Toplis R. Students’ views about secondary school science lessons: The role of practical work. Res Sci Educ. 2012;42: 531–549.

[9] Hampden-Thompson G, Bennett J. Science teaching and learning activities and students’ engagement in science. Int J Sci Educ. 2013;35: 1325–1343.

[10] Usher EL, Pajares F. Sources of self-efficacy at school: critical review of the literature and future directions. Rev Educ Res. 2008;78: 751–796.

[11] Zeldin AL, Britner SL, Pajares F. A comparative study of the self-efficacy beliefs of successful men and women in mathematics, science, and technology careers. J Res Sci Teach. 2008;45: 1036–1058.

[12] Pedaste M, Mäeots M, Siiman LA, de Jong T, van Reisen SAN, Kamp ET, et al Phases of inquiry-based learning: Definitions and the inquiry cycle. Educ Res Rev. 2015;14: 47–61.

[13] Falloon G. Forging school-scientist partnerships: A case of easier said than done? J Sci Educ Technol. 2014;22: 858–876.

[14] Hebert PDN, Cywinska A, Ball SL, deWaard JR. Biological identifications through DNA barcodes. Proc R Soc Lond B. 2003;270: 313–320. 10.1098/rspb.2002.2218 PMC1691236 12614582

[15] Bickford D, Lohman DJ, Sodhi NS, Ng PKL, Meier R, Winker K, et al Cryptic species as a window on diversity and conservation. Trends Ecol Evol. 2007;22: 148–155. 10.1016/j.tree.2006.11.004 17129636

[16] Blackett MJ, Semeraro L, Malipatil MB. Barcoding Queensland fruit flies (*Bactrocera tryoni*): impediments and improvements. Mol Ecol Resour. 2012;12: 428–436. 10.1111/j.1755-0998.2012.03124.x 22369549

[17] Rees HC, Maddison BC, Middleditch DJ, Patmore JRM, Gough KC. The detection of aquatic animal species using environmental DNA–a review of eDNA as a survey tool in ecology. J Appl Ecol. 2014;51: 1450–1459.

[18] Kane DE, Hellberg RS. Identification of species in ground meat products sold on the U.S. commercial market using DNA-based methods. Food Control. 2016;59: 158–163.

[19] Henter HJ, Imondi R, James K, Spencer D, Steinke D. DNA barcoding in diverse educational settings: Five case studies. Phil Trans R Soc B 2016;371: Article Number 20150340. 10.1098/rstb.2015.0340 PMC4971192 27481792

[20] Santschi L, Hanner RH, Ratnasingham S, Riconscente M, Imondi R. Barcoding life’s matrix: Translating biodiversity genomics into high school settings to enhance life science education. PLOS Biol. 2013;11: e1001471 10.1371/journal.pbio.1001471 23382648 PMC3558426

[21] Tosh J, James K, Rumsey F, Crookshank A, Dyer R, Hopkins D. Is DNA barcoding child’s play? Science education and the utility of DNA barcoding for the discrimination of tree species. Bot J Linn Soc. 2016;181: 711–722.

[22] Ainley J, Kos J, Nicholas M. Participation in science, maths and technology education in Australian education. 2008 Available at: http://research.acer.edu.au/acer_monographs/4/.

[23] Duncan J, Chiovitti T. Inspiring the next generation of immunologists. Eur J Immunol. 2016;46: 2688–2692.

[24] Chiovitti A, Duncan JC, Jabbar A. Promoting science in secondary school education. Trends Parasitol. 2017;33: 416–420. 10.1016/j.pt.2017.02.003 28274801

[25] Godin B, Schauz D. The changing identity of research: A cultural and conceptual history. Hist Sci. 2016;54: 276–306.

[26] Brownell SE, Price JV, Steinman L. Science communication to the general public: Why we need to teach undergraduate and graduate students this skill as part of their formal scientific training. J Undergrad Neurosci Educ. 2013; 12: E6–E10. 24319399 PMC3852879

[27] Clark G, Russel J, Enyeart P, Gracia B, Wessel A, Jarmoskaite I, et al (2016) Science educational outreach programs that benefit students and scientists. PLOS Biol. 2016;14: e1002368 10.1371/journal.pbio.1002368 26844991 PMC4742226

[28] Cleveland LM, Reinsvold RJ. Development of oral communication skills by undergraduates that convey evolutionary concepts to the public. J Microbiol Biol Educ. 2017;18: 1–4. 10.1128/jmbe.v18i1.1227 PMC5410759 28512518

[29] Nagy ZT, Sonet G, Glaw F, Vences M. First large-scale DNA barcoding assessment of reptiles in the biodiversity hotspot of Madagascar, based on newly designed CO1 primers. PLOS One. 2012;7: e34506 10.1371/journal.pone.0034506 22479636 PMC3316696

[30] Chapple DG, Ritchie PA. A retrospective approach to testing the DNA barcoding method. PLOS One 2013;8: e77882 10.1371/journal.pone.0077882 24244283 PMC3823873

[31] Jeong TJ, Jun J, Han S, Kim HT, Oh K, Kwak M. DNA barcode reference data for the Korean herpetofauna and their applications. Molec Ecol Resour. 2013;13: 1019–1032. 23311467 10.1111/1755-0998.12055

[32] Chambers EA, Hebert PDN. Assessing DNA barcodes for species identification in North American reptiles and amphibians in natural history collections. *PLOS One* 2016;11: e0154363 10.1371/journal.pone.0154363 27116180 PMC4846166

[33] Hawlitschek O, Morinière J, Dunz A, Franzen M, Rödder D, Glaw F, et al Comprehensive DNA barcoding of the herpetofauna of Germany. Molec Ecol Resour. 2016;16: 242–253. 25892157 10.1111/1755-0998.12416

[34] Vasconcelos R, Montero-Mendieta S, Simó-Riudalbas M, Sindaco R, Santos X, Fasola M, et al Unexpectedly high levels of cryptic diversity uncovered by a complete DNA barcoding of reptiles of the Socotra Archipelago. PLOS One. 2016;11: e0149985 10.1371/journal.pone.0149985 26930572 PMC4772999

[35] Chapple DG, Keogh S, Hutchinson MN. Substantial genetic substructuring in southeastern and alpine Australia revealed by molecular phylogeography of *Egernia whitii* (Lacertilia: Scincidae) species group. Molec Ecol. 2005;14: 1279–1292. 15813770 10.1111/j.1365-294X.2005.02463.x

[36] Endo Y, Nash M, Hoffmann AA, Slatyer R, Miller AD. Comparative phylogeography of alpine invertebrates indicates deep lineage diversification and historical refugia in the Australian Alps. J Biogeogr. 2015;42: 89–102.

[37] Lemke JL. Articulating communities: Sociocultural perspectives on science education. J Res Sci Teach. 2001;38: 296–316.

[38] Palmer D. A motivational view of constructivist-informed teaching. Int J Sci Educ. 2005;27: 1853–1881.

[39] Higgins DG, Bleasby AJ, Fuchs R (1992) Clustal V: improved software for multiple sequence alignment. Comput Appl Biosci. 1992;8: 189–191. 1591615 10.1093/bioinformatics/8.2.189

[40] Thompson JD, Higgins DG, Gibson TJ. CLUSTAL V: improving the sensitivity of progressive multiple sequence alignment through sequence weighting, position-specific gap penalties and weight matrix choice. Nucleic Acids Res. 1994;22: 4673–4680. 7984417 10.1093/nar/22.22.4673 PMC308517

[41] Felsenstein J. PHYLIP–Phylogeny Inference Package (Version 3.2). Cladistics. 1989;5: 164–166.

[42] Hall BG. Building Phylogenetic Trees from Molecular Data with MEGA. Molec Biol and Evol. 2103;30: 1229–1235. 10.1093/molbev/mst012 23486614

[43] Kumar S, Stecher G, Tamura K. MEGA7: Molecular Evolutionary Genetics Analysis version 7.0 for bigger datasets. Molec Biol and Evol. 2016;33: 1870–1874. 27004904 10.1093/molbev/msw054 PMC8210823

[44] Jukes TH, Cantor CR. Evolution of protein molecules In Munro HN, editor. Mammalian protein metabolism. New York: Academic Press; 1969 pp. 21–132.

[45] Geiger MF, Astrin JJ, Borsch T, Burkhardt U, Grobe P, Hand R, et al How to tackle the molecular species inventory for an industrialized nation–lessons from the first phase of the German Barcode of Life initiative GBOL (2012–2015). Genome. 2016;59: 661–670. 10.1139/gen-2015-0185 27314158

[46] Taraban R, Box C, Myers R, Pollard R, Bowen CW. Effects of active-learning experiences on achievement, attitudes and behaviours in high school biology. J Res Sci Teach. 2007;44: 960–979.

[47] Hulleman CS, Harackiewicz JM. Promoting interest and performance in high school science classes. Science. 2009;326: 1410–1412. 10.1126/science.1177067 19965759

[48] Pilot A, Bulte AMW. Why do you “Need to Know”? Context-based education. Int J Sci Educ. 2006;28: 953–956.

[49] Barak M. Science Teacher education in the twenty-first century: a pedagogical framework for technology-integrated social constructivism. Res Sci Educ. 2017;47: 283–303.

[50] Sarid A. Systematic thinking on dialogical education. Educ Philos Theory. 2012;44: 926–941.

[51] Tytler R, Osborne J. Student attitudes and aspirations towards science In Fraser BJ, Tobin KG, McRobbie CJ, editors. Second International Handbook of Science Education. Berlin: Springer; 2012 pp. 597–625.

[52] Hillman SJ, Bloodsworth KH, Tilburg CE, Zeeman SJ, List HE. K-12 students’ perceptions of scientists: Finding a valid measurement and exploring whether exposure to scientists makes an impact. Int J Sci Educ. 2014;36: 2580–2595.

[53] Woods-Townsend K, Christodoulou A, Rietdijk W, Byrne J, Griffiths JB, Grace MM. Meet the scientist: The value of short interactions between scientists and students. Int J Sci Educ Part B. 2016;6: 89–113.

[54] Bøe MV, Henrikson EK, Lyons T, Schreiner C. Participation in science and technology: Young people’s achievement-related choices in late-modern society. Stud Sci Educ. 2011;47: 37–72.

[55] Bonney R, Phillips TB, Ballard HL, Enck JW. Can citizen science enhance public understanding of science? Public Underst Sci. 2016;25: 2–16. 10.1177/0963662515607406 26445860

[56] Zoellick B, Nelson SJ, Schauffler M. (2012) Participatory science and education: bringing both views into focus. Front Ecol Environ. 2012;10: 310–313.

