# Supplementary material for: A citizen science model for implementing statewide educational DNA barcoding
Source: PLoS One. 2019 Jan 10;14(1):e0208604. doi: 10.1371/journal.pone.0208604 (PMC6328199; doi:10.1371/journal.pone.0208604)
Supplement: S1 Table — (PDF) [file pone.0208604.s001.pdf]

S1 Table. Collection data for specimens investigated in the project with attributions to contributing students and PhD scientist mentors.

MV Rego #	Voucher specimen	Common name	Scientific name	Collection date	Locality	Genbank accession number	Contributors
**Z22563**	D75424	Eastern three- lined skink	*Acritoscincus duperreyi*	22/11/2012	Black Range State Forest, Muirfoot Track,	n/a	K. Meiselbach [1], M. Danaro [1], T. Johanson [1], S. Cassim [1], B. Luke [14], A. Barimah [14], B. Harris [6], E. Hunt [6], H. Dawes-Robb [9], D. Samuel [9], A. Koubar [12]
**Z22574**	D75418	Eastern three- lined skink	*Acritoscincus duperreyi*	22/11/2012	Grampians National Park, The Pinnacle and Sundial Peak Picnic Area	n/a	M. Pert [1], M. Danaro [1], F. Angrisano [1], S. Chockalingam [1], E. Callanan [14], C. Hollingworth [14], J.-L. Ross [6], K. Elliott [6], J. Jones-Rolls [9], C. Puyol [9], Linh [12], Julie [12]
**Z22402**	D75343	Jacky dragon	*Amphibolurus muricatus*	19/11/2012	Grampians National Park, Jananginj Njaui Track, 1.7-2.5km by road W of intersection with Red Rock Road	n/a	K. Meiselbach [1], M. Danaro [1], S. Longmuir [1], S. Han [1], J. Verity [14], J. Rice [14], M. Deckman [6], M. Lochhead [6], M. Harris [9], F. Krucers-Ozolins [9], L. Bolitho [16], T. Robson [16]
**Z22414**	D75337	Jacky dragon	*Amphibolurus muricatus*	19/11/2012	Grampians National Park, intersection of Syphon Road and Glenelg River	n/a	S. Walia [1], F. Angrisano [1], G. Jones [1], J. Scanlan [1], J. Kolanowski [6], N. Evans [6], D. Hopkins [9], N. Le [9], Jennifer [9], Tien [9], R. Redpath [16], S. Steinkellner [16]
**Z27172**	D75634	Jacky dragon	*Amphibolurus muricatus*	Nov-13	Alpine National Park, Cobberas track	MH028635	S. Ostrouska [1], S. Longmuir [1], A. Cutting [1], Y. Pace [1], M. Tucker [3], M. Karslake [3], C. Willie [12], J. Lay [12], J. Rasha [12], D. Ali Ahmad [12], E. Robinson [10], K. Croft [10]
**Z29155**	D75668	Jacky dragon	*Amphibolurus muricatus*	Nov-13	Alpine National Park, Beloka Road Granite Outcrop	MH028622	J. Scanlan [1], S. Han [1], B. Wood [1], S. Ostrouska [1], J. Maori [4], T. Tran [4], R. Gonato [7], C. Barn Father [7], J. Vasilakis [17], J. Crawford [17]

**Z22340**	D75403	Marbled Gecko	*Christinus marmoratus*	21/11/2012	Grampians National Park, Jananginj Njaui Track	n/a	S. Walia [1], S. Chau [1], A. Chowdhury [1], R. Vanstone [6], K. Fleeton [6], M. Halfyard [13], M. Scanlon [13], A. Radoja [12], J. Pernice [12]
**Z22408**	D75331	Marbled Gecko	*Christinus marmoratus*	18/11/2012	Grampians National Park, Cooinda Burrong Scout Camp	n/a	E. Zuccala [1], S. Moneer [1], C. Abbott [1], C. Douglas [6], S. Peckham [6], P. Cerdei [11], A. Finne-Larson [11], D. Lora [12], N. Banney [8]
**Z22533**	D75444	Fence skink	*Cryptoblepharus pannosus*	24/11/2012	Grampians National Park, area surrounding Mount Zero Picnic Area and Hollow Mountain car park	n/a	J. Cuxson [1], H. Vanyai [1], A. Lun [1], L. Wiratno [8], N. Valeska [8], S. Zhong [5], L. Awad [5], L. Nguyen [7], D. Simpson [7]
**Z22535**	D75446	Fence skink	*Cryptoblepharus pannosus*	24/11/2012	Grampians National Park, area surrounding Mount Zero Picnic Area and Hollow Mountain car park	n/a	K. Meiselbach [1]. S. Moneer [1], S. Cassim [1], S. Canham [14], A. Lapojapo [11], C. Amum [11], J. Tomas [11], J. Dang [12]
**Z22519**	D75465	Robust skink/ Eastern striped skink	*Ctenotus robustus*	24/11/2012	Grampians National Park, area surrounding Mount Zero Picnic Area and Hollow Mountain car park	n/a	J. Cuxson [1], S. Ostrouska [1], C. Diaz [1], L. McLachlan [8], N. Jones [8], David [12], Leslie [12], T. McLean-Ingleton [17], B. Forrester [17]

**Z22531**	D75459	Robust skink/ Eastern striped skink	*Ctenotus robustus*	24/11/2012	Grampians National Park, area surrounding Mount Zero Picnic Area and Hollow Mountain car park	n/a	M. Pert [1], E. Zuccala [1], S. Longmuir [1], B. Dooley [14], M. Lamb [14], L. Matthias-Williamson [6], J. Weller [6], E. Trethewey [9], N. Gaoulil [9]
**Z27200**	D75661	Copper tailed skink	*Ctenotus taeniolatus*	Nov-13	Alpine National Park, Beloka Road Granite Outcrop	n/a	S. Chau [1], A. Nguyen [1], H. McRae [1], S. Preston [1], N. Fisher [3], T. D'Ombrain [3], I. Zhimomi [10], S. Prakash [7], K. Cousevska [7], A. Robinson [19], C. Robinson [19]
**Z27235**	D75698	Copper tailed skink	*Ctenotus taeniolatus*	Nov-13	Alpine National Park, Beloka Road Granite Outcrop	n/a	K.J. Tan [1], S. Belluzzo [1], H. McRae [1], S. Ostrouska [1], E. Cameron [3], G. Gibb [3], S. Kulg [12], R. Nettasingha [12], C. Anderson [19], A. Walters [19], B. Dewhurst [17], B. Gibbons [17]
**Z29153**	D75666	Copper tailed skink	*Ctenotus taeniolatus*	Nov-13	Alpine National Park, Beloka Road Granite Outcrop	n/a	N. Donker [1], A. Cutting [1], S. Longmuir [1], L. Green [1], A. McDonald [3], J. Mirabella [3], A. Le [1], T. Auvale [1], L.N.T. Bawi [4], K. Kediye [4], F. Inguillo [17], C. Lees [17]
**Z29154**	D75667	Copper tailed skink	*Ctenotus taeniolatus*	Nov-13	Alpine National Park, Beloka Road Granite Outcrop	n/a	A. Nguyen [1], J. Hawkey [1], N. Donker [1], S. Nguyen [4], B. Huynh [4], J. Patton [7], S.-L. Missio [7], C. Cutrupi [7], S. McRostie [19], J. Rainbow [19]
**Z29307**	D75731	Copper tailed skink	*Ctenotus taeniolatus*	Nov-13	Alpine National Park, Snowy River Road	MH028632	L. Green [1], J. Hawkey [1], Y. Pace [1], S. Chau [1], D. Kaur [12], A, Nguyen [12], M. Noori [10], A. Miller [17], S. Jacobs [17]
**Z27192**	D75654	Cunningham's skink	*Egernia cunninghami*	Nov-13	Alpine National Park, Beloka Road Granite Outcrop	MH028630	S. Moneer [1], S. Ostrouska [1], S. Longmuir [1], S. Han [1], A. Cutting [1], N. Donker [1], Z. Mousaco [1], R. Meuwly [1], L. Nguyen [12], K. Cheng [12], N. Anuwae [4], A. Nguyen [4], J. Frank [4], C. Dut [4], C. Plant [15], T. MacDonald [15], M. Riccardi [7], S. Muraru [7]
**Z22514**	D75476	Black rock skink	*Egernia saxatilis*	24/11/2012	Grampians National Park, Tower Hill	n/a	E. Zuccala [1], T. Johanson [1], H. Eason [1], K.J. Tan [1], Ann [1], J. Newcombe [6], T. Straw [6], I. Rusker [9], S. Kydas [9], J. Aguiar [8], E. Barthe [8], C. Carbone [16], J. Chew [16], B. Aprem [7], T. Knight [7]

**Z22515**	D75461	Black rock skink	*Egernia saxatilis*	24/11/2012	Grampians National Park	n/a	S. Chau [1], S. Cassim [1], G. Jones [1], H. Vanyai [1], J. Koffeyberg [13], Z. Mihan [13], T. Tran [12], B. Kohr [12], Shelly [12], Jenny T. [12], F. Anderson-Cleary [5], L. Fisher [5]
**Z26887**	D75751	Black rock skink	*Egernia saxatilis intermedia*	Nov-13	Alpine National Park, Ramshorn peak to carpark and moth cave	MH028629	L. Green [1], S. Ostrouska [1], B. Wood [1], A. Lun [1], D. Nguyen [12], N. Phan [12], M. Mazzeil [7], B. Morales [7], M. Vang [7], C. Collett [17], M. Walkden [17]
**Z26888**	D75751	Black rock skink	*Egernia saxatilis intermedia*	Nov-13	Alpine National Park, Ramshorn peak to carpark and moth cave	MH028629	K. Rankin [1], P. Gradie [1], S. Belluzzo [1], U. Chandrasiri [1], J. Cartes [10], S. Bedak [10], B. Shaikh [7], M. Iberras [7], T. Rowlands [17], D. Greenfield [17]
**Z26891**	D75754	Black rock skink	*Egernia saxatilis intermedia*	Nov-13	Alpine National Park, Ramshorn peak to carpark and moth cave	MH028609	S. Ostrouska [1], S. Han [1], K.J. Tan [1], B. Wood [1], K. Leckie [3], E. Grace [3], D. Smilek [1], V. Pham [4], J. Nguyen [4], C. Benstead [19], R. Boyd [19]
**Z29306**	D75730	Black rock skink	*Egernia saxatilis intermedia*	Nov-13	Alpine National Park, Limestone Road, waterfall	MH028628	S. Belluzzo [1], A. Nguyen [1], S. Preston [1], J. Tcet [12], W. Nguyen [12], S. Webber [10], H, Simon [19], T. Langborne [19]
**Z27240**	D75700	Black rock skink	*Egernia saxatilis intermedia*	Nov-13	Alpine National Park, Rams Horn	MH028617	S. Preston [1], S. Moneer [1], J. Hawkey [1], H. McRae [1], M. Kawa [3], T. Marnsey [3], S. Cleary [1], S. Deev [1], C. Trinh [4], A. Thang [4], J. Corsetti [7], D. Arena [7]
**Z22407**	D75347	Southern water skink	*Eulamprus tympanum tympanum*	19/11/2012	Grampians National Park, Victoria Range Track	n/a	H. Eason [1], S. Chockalingam [1], S. Polling [1], K.J. Tan [1], U. Chandrasiri [1], A. Lun [1], J. Stratton [1], E. Vergnano [8], F. Sampaio [8], Tracy [12], Helena [12], James [12], Terri [12], S, Manniche [16], B. Gregoire [16], J. Pentland [5], H. De Oliviera [5], C. Forth [7], K. Kassar [7], C. Ignatiaois [2], D. Kaldas [2]
**Z22529**	D75458	Southern Water skink	*Eulamprus tympanum tympanum*	24/11/2012	Grampians National Park, Golten Gorge Picnic Area	n/a	S.Chau [1], M. Lau [1], S. Han [1], B. Steed [1], R. O'Regan [9], M. Manteit [9], Joanna [12], Kathy [12], C. Parus [16], M. O'Sullivan [16], G. Radojcic [7], G. Vervoort [7]

**Z22546**	D75439	Southern Water skink	*Eulamprus tympanum tympanum*	24/11/2012	Grampians National Park, Roses Gap, Beehive Falls	n/a	D. Yilmaz [1], S. Longmuir [1], C. Abbott [1], H. Vanyai [1], J. Stratton [1], A. Harper [6], D. Simpson [6], J. Claire [9], J. Bennet [9], J. Nguyen [12], J. Ly [12], E. Vatcky [2], S. Thanaskanda [2]
**Z26840**	D75592	Southern water skink	*Eulamprus tympanum tympanum*	Nov-13	Alpine National Park, road to Rams Horn 3	MH028627	U. Chandrasiri [1], K. Rankin [1], A. Nguyen [1], L. Green [1], D. Leiskie [17], T. Gatkuoth [10], E. Fletcher-Lyle [17], H. Robertson [17]
**Z27151**	D75596	Southern water skink	*Eulamprus tympanum tympanum*	Nov-13	Alpine National Park, Native Dog Flat	MH028633	W.J. Scanlan [1], B. Wood [1], L. Green [1], R. Golaw [7], T. Waqalevu [7], K. Shawcroft [17], R. Dean [17]
**Z27153**	D75598	Southern water skink	*Eulamprus tympanum tympanum*	Nov-13	Alpine National Park, Rocky Plain Creek, off Limestone Creek	MH028623	S. Chau [1], S. Ostrouska [1], B. Steed [1], B. Wood [1], N. Lewis [3], S. Pollard [3], E. Akkaplan [12], D. Nguyen [12], F. Uraha [7], M. Lambardo [7], C. Treven [19], S. Cornish [19]
**Z27158**	D75603	Southern water skink	*Eulamprus tympanum tympanum*	Nov-13	Alpine National Park, creek crossing below Davies Plain Hut Campground	MH028619	P. Gradie [1], S. Han [1], A. Cutting [1], S. Preston [1], M.-T. Nguyen [12], N. Astono [12], K. Nguyen [4], K. Tran [4], J. Parker [15], E. Garrett [15], A. Quigley [15], S. Struc [19], K. Birch [19], A. Banks [19]
**Z29168**	D75723	Southern water skink	*Eulamprus tympanum tympanum*	Nov-13	Alpine National Park, Limestone Road, Bouyard Creek crossing	MH028618	S. Preston [1], Y. Pace [1], J. Scanlan [1], E. Matheson [3], J. Burton [3], S. Nicholas [1], L. Vavvados [1], E. Ayres [4], M. Demirovic [4]
**Z22393**	D75353	Grass skink	*Lampropholis guichenoti*	20/11/2012	Grampians National Park, The Pinnacle and Sundial Peak Picnic Area	n/a	A. Chowdhury [1], A. Tokanovic [1], C. Valkovic [12], T. Read [12]
**Z27144**	D75585	Grass skink	*Lampropholis guichenoti*	Nov-13	Alpine National Park, Limestone	MH028610	S. Ostrouska [1], S. Longmuir [1], K. Rankin [1], A. Nguyen [1], G. Howell [3], E. Thompson [3], A. Catic [12], R. Mansour [12], Hanaira [10], A. De Jong [19], B. Smith [19]

					Road, Native Dog Flat Campground		
**Z29163**	D75719	Grass skink	*Lampropholis guichenoti*	Nov-13	Alpine National Park, Bulley Creek at Cowombat Track	MH028634	A. Nguyen [1], J. Hawkey [1], H. McRae [1], S. Chau [1], N. Nuynh [4], T. Nguyen [4], L, Dunleavy [19], J. Wynn [19], S. Murnane [17], D, Hughes [17], O. Peacock [17]
**Z29165**	D75725	Grass skink	*Lampropholis guichenoti*	Nov-13	Alpine National Park, Rocky outcrop on Cowombat Track	MH028611	N. Donker [1], Y. Pace [1], P. Gradie [1], U. Chandrasiri [1], A. Wiltshire [3], E. Werts [3], J. Amanda [1], Y. Duong [1], P. Marriott [10], J. Raqiza [10], F. Faiyaz [7], F. Yousif [7]
**Z29305**	D75729	Grass skink	*Lampropholis guichenoti*	Nov-13	Alpine National Park, Limestone Road, waterfall	MH028624	L. Green [1], J. Hawkey [1], B. Steed [1], S. Ostrouska [1], N. Nguyen [12], J. Niaz [12], M. Benson [7], K. Wild [7], D. Gihooley [17]
**Z29309**	D75734	Grass skink	*Lampropholis guichenoti*	Nov-13	Alpine National Park, McFarlane Flat Track, Berrima Creek crossing	MH028620	S. Han [1], W.J. Scanlan [1], A Cutting [1], K. Isiah [1], I. Houssein [4], M. Huynh [4], A. Coates [15], E. Norris [15]
**Z22375**	D75369	no common name	*Lerista bougainvillii*	21/11/2012	Grampians National Park, The Pinnacle and Sundial Peak Picnic Area	n/a	S. Ostrouska [1], A. Tokanovic [1], K. Brovedani [1], Pat [12], Ken [12], G. Anderson [5], N. Davis [5], D. Pluckrose [18], L. Woods [18]
**Z22376**	D75370	no common name	*Lerista bougainvillii*	21/11/2012	Grampians National Park, The Pinnacle and Sundial Peak Picnic Area	n/a	G. Jones [1], C. Diaz [1], Kathy [12], Jenny N [12], J. Li [16], A. Jaros [16]
**Z22552**	D75431	Eastern Banjo Frog	*Limnodynastes dumerilii*	23/11/2012	Black Range State Forest, Muirfoot Track	n/a	C. Abbott [1], S. Polling [1], R. Manhire-Heath [1], B. Steed [1], Ann [1], E. Moss [1], M. Roberts [1], Karren [1], Nikki [1], L . Palmarella [1], C. Goodman [1], M. Bevis [1], R. Cannon [1], K. Wappett [1]

**Z22567**	D75428	Eastern Banjo Frog	*Limnodynastes dumerilii*	23/11/2012	Black Range State Forest, Muirfoot Track	n/a	S. Han [1], J. Scanlan [1], A. Lun [1], B. Steed [1], B. Carrucan [16], G. Duffy [16], K. Forest [16], K. Myles [16], S. Kumar [7], M. Elias [7], P. Pastrikos [7], S. Masoe [7]
**Z22378**	D75372	White's skink	*Liopholis whitii*	21/11/2012	Grampians National Park, The Pinnacle and Sundial Peak Picnic Area	n/a	T. Johanson [1], S. Chockalingam [1], R. Manhire-Heath [1], J. Stratton [1], B. Allen [9], G. Bradford [9], Cindy [12], Jasmine [12], N. Petropoulos [5], M. Hamid [5], D. Paris [2], A. Molinaro [2]
**Z22379**	D75373	White's skink	*Liopholis whitii*	21/11/2012	Grampians National Park, The Pinnacle and Sundial Peak Picnic Area	n/a	D. Yilmaz [1], S. Moneer [1], A. Chowdhury [1], C. Diaz [1], A. Allan [6], G. Leeson [6], D. Tan [11], K. Duniter [11], N. Wilkolawski [12], D. Alispahic [12], M. Kelly [16], C. Sharp [16]
**Z22520**	D75466	no common name	*Morethia boulengeri*	24/11/2012	Grampians National Park, area surrounding Mount Zero Picnic Area and Hollow Mountain car park	n/a	M. Lau [1], A. Tokanovic [1], Truong [12], Samiha [12], G. Flanagan [5], A. Constable [5]
**Z22558**	D75434	no common name	*Morethia boulengeri*	24/11/2012	Grampians National Park, area surrounding Mount Zero Picnic Area and Hollow Mountain car park	n/a	F. Angrisano [1], K. Brovedani [1], A. Davislim [9], T. Tran [9], A. Campbell [18], E. Ellis [18]
**Z27130**	D75888	Tussock skink	*Pseudemoia entrecasteauxii*	Nov-13	Alpine National Park, road to Rams Horn 3	MH028613	K.J. Tan [1], P. Gradie [1], Y. Pace [1], H. McDonough [3], J. Featherstone [3], K. Lee [12], M.-A. Ho [12], L. Aylin [12], V. Ibrahim [12], D. Hayes [10], J. Welbourne [10]

**Z27145**	D75586	Tussock skink	*Pseudemoia entrecasteauxii*	Nov-13	Alpine National Park, Rams Horn Track	MH028621	A. Nguyen [1], K.J. Tan [1], U. Chandrasiri [1], H. McRae [1], P. Tran [4], N. Mani [7], J. Smith [7], G. Garwood [19], K. Triffett [19]
**Z27154**	D75599	Tussock skink	*Pseudemoia entrecasteauxii*	Nov-13	Alpine National Park, Rocky Plain Creek	MH028625	W.J. Scanlan [1], Y. Pace [1], S. Belluzzo [1], A. Dornish [10], F. To [4], Y. Hagi [4], N. Watson [7], K. Broder [7]
**Z27182**	D75644	Tussock skink	*Pseudemoia entrecasteauxii*	Nov-13	Alpine National Park, Limestone Road, Native Dog Flat Campground	MH028612	S. Chau [1], A. Cutting [1], B. Steed [1], I.S. Paul [3], J. Stewart [3], E. Nada [1], C. Petricevic [1], R. Medina [7], S. Eshow [7], J. Geer [17], J. Wynne [17]
**Z27232**	D75696	Tussock skink	*Pseudemoia entrecasteauxii*	Nov-13	Alpine National Park, Davies Plain track	MH028631	S. Belluzzo [1], K. Rankin [1], A. Nguyen [1], A. Lun [1], K. Nguyen [12], T. La [12], Shaz [10], Sarah [10], J. Brooks [19], S. May [19], H. Smith [19], J. Lothian [17], F. MacDonald [17]
**Z27139**	D75897	Mountain dragon	*Rankinia diemensis*	19/11/2013	Victoria, Alpine National Park, Rams Horn Track	MH028615	S. Preston [1], K. Rankin [1], H. McRae [1], N. Donker [1], A. Pan [3], K. Agraviador [3], Aiden [10], Miles [10], T. Frazier [7], A. Brewster [7], R. Currie [7], A. Holmberg [7]
**Z27169**	D75631	Mountain dragon	*Rankinia diemensis*	Nov-13	Alpine National Park, road to Rams Horn 3	MH028614	N. Donker [1], K.J. Tan [1], U. Chandrasiri [1], B. Wood [1], R. Jennings [3], B. Douglass [3], K. Lam [4], E. Chu [4], M. Sumperos [7], J. Totino [7], B. Slade [19], D. Berdilek [19]
**Z27171**	D75633	Mountain dragon	*Rankinia diemensis*	Nov-13	Alpine National Park, Cowombat track near Murray River	MH028616	K.J. Tan [1], P. Gradie [1], S. Han [1], J. Smart [3], A. Stones [3], J. Pinijaram [12], T. Lam [12], B. Fernando [1]
**Z27183**	D75645	Mountain dragon	*Rankinia diemensis*	Nov-13	Alpine National Park, Cowombat Flat Track	MH028626	K. Rankin [1], S. Belluzzo [1], A. Lun [1], U. Chandrasiri [1], J. Duell [1], D. Goodall [1], E. Dixon-Sole [1], A. Hinton [1], L. Smith [1], J. Knowles [1]
**Z27196**	D75655	Blotched bluetongue lizard	*Tiliqua nigrolutea*	Nov-13	Alpine National Park, Wambargo Range, Limestone Road	n/a	S. Moneer [1], J. Hawkey [1], S. Longmuir [1], M. Flamenco [1], K. Tavazina [1], S. Nguyen [12], Q. Nguyen [12], S. van Staden [12], J. Tsemetzis [12], N. Ma [4], L.H. Par [4]
**Z22404**	D75344	Shingleback, Sleepy Lizard	*Tiliqua rugosa*	19/11/2012	Grampians National Park, intersection of	n/a	M. Pert [1], S. Walia [1], H. Eason [1], J. Cuxson [1], S. Ostrouska [1], M. Lau [1], S. Polling [1], J. Scanlan [1], U. Chandrasiri [1], Ann [1], K. Brovedani [1], O. Jewers-

					Syphon Road and Glenelg River		Donohoe [14], T. Fleeton [6], M. Nicholls [6], A. Miaskovski [8], S. Kuttner [8], S. Ozgur [8], J. Moore [8], Rabia [12], Hainoame [12], Peumi [12], Louise [12], Jaspeet [12], Steph [12], M. Selwood [16], K. Southam [16], M. Thavarajah [5], G. Ryan [5], J. Atwa [2], Y. Karabatsos [2], S. Weiss-O'Leary [7], S. Peell [7]

[1] GTAC scientist mentor, [2] Alphington Grammar School, [3] Ballarat High School, [4] Braybrook Secondary College, [5] Brunswick Secondary College, [6] Castlemaine Secondary College, [7] Craigieburn Secondary College, [8] Elwood College, [9] Fitzroy High School, [10] Hallam Senior Secondary College, [11] Heritage College, [12] Keilor Downs Secondary College, [13] Lilydale Adventist Academy, [14] McClelland College, [15] Neerim District Secondary College, [16] Sacred Heart College, [17] Sandringham Secondary College, [18] Upper Yarra Secondary College, [19] Wallan Secondary Colleg
